# A Simple Surgical Approach for the Management of Acquired Severe Lower Punctal Stenosis

**DOI:** 10.1155/2019/3561857

**Published:** 2019-01-14

**Authors:** Sameh S. Mandour, Khaled E. Said-Ahmed, Hany A. Khairy, Moataz F. Elsawy, Marwa A. Zaky

**Affiliations:** Department of Ophthalmology, Menoufia University, Faculty of Medicine, Shebin El Kom, Menoufia, Egypt

## Abstract

**Purpose:**

Evaluation of using pigtail probe to detect and open severely stenosed lower lacrimal punctum followed by self-retaining bicanalicular intubation.

**Study design:**

A prospective nonrandomized clinical study.

**Methods:**

The study included 24 patients with severe lower punctal stenosis (grade 0 according to Kashkouli scale) attending at Menoufia University Hospitals. The upper punctum and canaliculus were patent. All patients were complaining of epiphora and had a thorough ophthalmological examination including dye disappearance test and slit-lamp examination. Pigtail probe was used from patent upper punctum to detect the lower stenosed punctum which was opened with a scalpel. Syringing of the lower lacrimal passages was done to confirm its patency, and self-retaining silicone bicanalicular stent was inserted. The silicone tube was left in place for 6 months before it was removed. Patients were then followed-up for 1 year after the surgery.

**Results:**

One year after surgery, epiphora was absent (grade 0) in 16 eyes (66.7%) and was present only occasionally (grade 1) in 4 eyes (16.7%). The difference from preoperative epiphora was statistically significant. One year after surgery, fluorescein dye disappearance time was grade 1 (<3 minutes) in 20 cases (83.3%), and grade 2 (3–5 minutes) in 4 cases (16.7%). There was a statistically significant difference compared with preoperative results.

**Conclusion:**

Using the pigtail probe is effective in treatment of severe punctal stenosis. Maintaining the punctal opening and prevention of restenosis can be achieved by using self-retaining bicanalicular stent after confirmation of nasolacrimal duct patency. This trial is registered with NCT03731143.

## 1. Background

Acquired external punctal stenosis (AEPS) may result from cicatricial conjunctival disease, chemical burn, herpetic viral infection, toxic effects of topical or systemic medications, various infections, eyelid malposition, aggressive lacrimal probing, or different forms of trauma and tumors. Aging changes can also cause punctal stenosis [1].

Currently, several procedures have been evolved to treat punctal stenosis [2–10]. However, all depends on the presence of a landmark for the stenosed punctum. In many instances, it is very difficult to recognize the site of the stenosed punctum which add a considerable challenge to the case. The pigtail probe has generally been used for treatment of canaliculus lacerations [11, 12]. Recently, it was used to detect the site of the occluded punctum in a small case series and proved effective [13].

In the current study, the authors used the pigtail probe to detect the site of a severely stenosed punctum followed by self-retaining bicanalicular intubation of the upper and lower puncti and canaliculi.

## 2. Patients and Methods

This is a prospective nonrandomized study which was conducted upon 24 patients with total lower punctual occlusion attending at Menoufia University Hospitals in the period from January 2014 to January 2018. Ethics approval from the institutional review board was obtained, and a written informed consent was taken from every patient according to the Declaration of Helsinki.

Inclusion criteria included the following: (1) severe lower punctual stenosis (grade 0 according to Kashkouli et al.'s scale of punctal stenosis (Table 1) [14]), (2) patent upper punctum and canaliculus as well as patent nasolacrimal duct as proved by probing and syringing through upper patent punctum, and (3) normal lower eyelid margin position.

Exclusion criteria included patients with punctal stenosis (grades more than 0 according to Kashkouli scale), patients with previous eyelid surgery, or patients with a lump overlying or involving the punctum or other part of the tear drainage system. And we excluded cases with lower canalicular or common canalicular stenosis or obstruction that were discovered intraoperatively. In these cases, the pigtail probe could not pass easily to the lower canaliculus and punctum, so they were shifted to another surgical plan as conjunctivodacryocystorhinostomy and Jones tube insertion.

All patients of the study were complaining of epiphora and had a thorough ophthalmological examination including fluorescein dye disappearance (FDD) test and slit-lamp examination. The dye disappearance test was performed with a drop of 2% fluorescein sodium dye in the inferior fornix. Assessment after 5 minutes of the remaining dye in the tear meniscus was done, and results were graded (Table 2) [15].

## 3. Surgical Procedure

All operations were done under general anesthesia and were performed by two authors (SSM, KES). The authors performed lacrimal probing and syringing test through the normal punctum to exclude concomitant occluded common canaliculus or nasolacrimal duct. The pigtail probe was passed through the canalicular system from the normal punctum to the occluded aspect. If the pigtail probe could not pass to the lower punctal site, an obstruction at the level of lower or common canaliculus was inspected, and the case was shifted to another surgical plan and accordingly excluded from the study.

**Figure 1:**
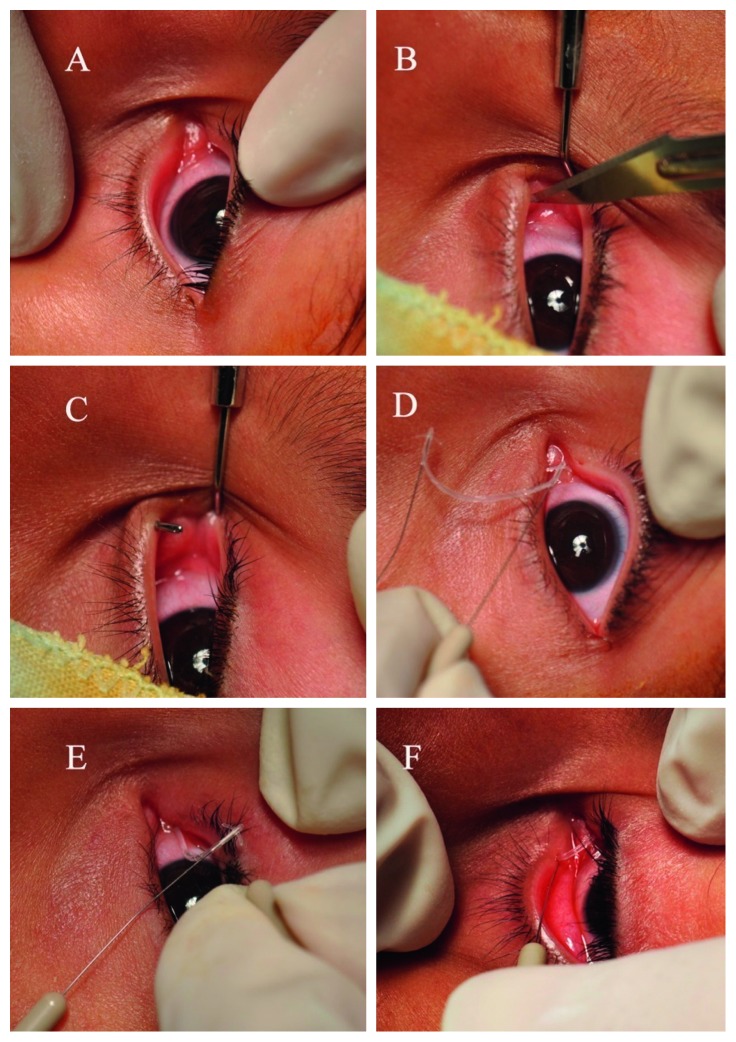
(a) Appearance of grade 0 lower punctal stenosis. (b) Using the pigtail probe to detect the stenosed lower punctum. (c) Opening of the lower punctum. (d) Inserting the self-retaining bicanalicular stent through the patent upper punctum and canaliculus. (e) Inserting the self-retaining bicanalicular stent through the patent lower punctum and canaliculus. (f) The final appearance after insertion of the stent.

When the tip of the pigtail probe was positioned near the occluded punctal area, the surgeon pushed the area to be tented with the pigtail probe (Figure 1(b)). After they advanced the pigtail probe back and forth several times (until they could locate the correct position of the occluded punctum), the authors incised the tented area with a scalpel No. 11 to make a new punctal opening (Figure 1(c)).

**Figure 2:**
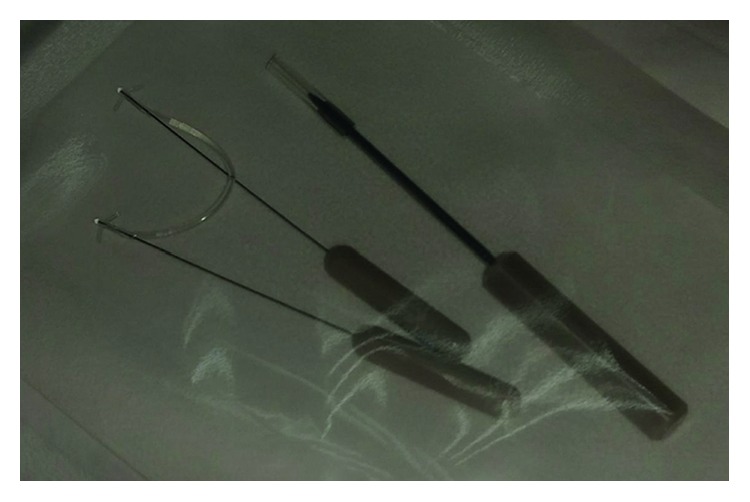
The self-retaining bicanalicular stent.

To ensure punctal and canalicular patency, syringing was repeated through the perforated punctum. To prevent reocclusion of punctal opening, a self-retaining bicanalicular tube (FCI®; Paris, France, Figure 2) was inserted through the normal and perforated puncti (Figures 1(d)–1(f)).

The silicone tube was left in place for 6 months before it was removed. Patients were then followed-up for 1 year after the surgery (6 months after removal of the tube). During the follow-up period, the authors investigated the improvement of subjective epiphora symptoms based on Munk score (Table 3) [16], fluorescein disappearance test, maintenance of newly formed punctal opening, and incidence of complications. The case was considered anatomically successful if the punctum maintained a Kashkouli score above 1. Functionally, complete success was achieved when Munk score was 0 or 1 and FDD score was 1. Partial success was achieved when Munk score was 2 and FDD score was 2. Failure was defined anatomically by persistent punctal stenosis (grades 0 or 1) and functionally by Munk score of more than 2 and FDD score of 3.

Statistical analysis was performed using SPSS version 16 (IBM corporation, Somers, NY) software. Paired *t*-test was used to detect the difference between pre- and postoperative data in the study group. Significance was considered at *p* < 0.05.

## 4. Results

This study included 24 eyes of 18 patients, 8 males and 10 females with a mean age of 44.71 ± 12.3 years (range, 17–67 years). All patients had punctal stenosis grade 0. Preoperative epiphora was grade 3 (epiphora requiring dabbing five to ten times per day) in 9 eyes (37.5%) and grade 4 (epiphora requiring dabbing more than ten times daily or constant tearing) in 15 eyes (62.5%). The preoperative fluorescein dye disappearance test was grade 1 (<3 minutes) in 3 eyes (12.5%), grade 2 (3–5 minutes) in 2 eyes (8.3%), and grade 3 (>5 minutes) in 19 eyes (79.2%).

Six months after surgery, epiphora was absent (grade 0) in 18 eyes (75%) and was present only occasionally (grade 1) in 3 eyes (12.5%). Patients (*N*=16) with grade 0 and 1 were satisfied with the result. One patient had one eye with grade 2 (4.2%), and one patient had his both eyes with grade 3 (8.3%). The difference from preoperative epiphora was statistically significant (*p* < 0.001) (Table 4).

One year after surgery (6 months after removal of the tube), epiphora was absent (grade 0) in 16 eyes (66.7%) and was present only occasionally (grade 1) in 4 eyes (16.7%). Patients (*N*=15) with grades 0 and 1 were satisfied. 2 eyes (8.3%) in 2 patients were grade 2 and 2 eyes (8.3%) of one patient were grade 3. The difference from preoperative epiphora was statistically significant (*p* < 0.001) (Table 4).

Six months after surgery, fluorescein dye disappearance time was grade 1 (<3 minutes) in 21 cases (87.5%) and grade 2 (3–5 minutes) in 3 cases (12.5%). There was a statistically significant difference compared with preoperative results (*p* < 0.001) (Table 5).

One year after surgery (6 months after removal of the tube), fluorescein dye disappearance time was grade 1 (<3minutes) in 20 cases (83.3%) and grade 2 (3–5 minutes) in 4 cases (16.7%). There was a statistically significant difference compared with preoperative results (*p* < 0.001) (Table 5).

Table 6 shows the difference in Kashkouli score among study patients 6 months and 12 months after the surgery. No case has returned to score 0 during the follow-up period. With regard to complications of surgery, 2 cases (8.3%) had extrusion of the tube before 6 months, where dilatation and reinsertion was done.

## 5. Discussion

Viso et al. [17] diagnosed punctal stenosis when a punctal opening could not be found under slit-lamp biomicroscopy and when a punctal dilator could not pass through the puncta. Kashkouli et al. [14] graded punctal stenosis from grade 0 (could not find papilla and punctum) to grade 5 (large punctal opening of greater than 2 mm) according to punctal sizes examined under slit-lamp biomicroscopy. In the authors' study, all patients were classified as grade 0 according to this scale.

Several procedures have been evolved to treat punctal stenosis [2–10]. Unfortunately, the position of the stenotic punctal opening should be present for those procedures to be done. However, in more severe cases in which it is difficult to find the punctal opening because of the severity of stenosis, it would be nearly impractical to proceed with these traditional methods. In a trial to overcome such difficult situations, a surgeon might try to guess the probable site of the occluded punctum puncturing it with punctum dilator or making an incision and dissection. That would create a false passage complicating the procedure with a very high risk of failure or recurrence even after a while of apparent success.

The pigtail probe in its original form had a sharp crochet hook on its tip to easily locate the proximal transected end of a lacerated canaliculus. However, this crochet hook turned out to have a destructive effect [18]. A currently widely used eyed pigtail probe with a round tip was developed by Beyer [19] in 1974. Jordan et al. [18] used this round-tipped, eyed pigtail probe and silicone tube together to reconstruct canalicular laceration in 228 patients, and he reported high success rate as 83.8%. A significant number of literatures have reported on the safety and the effectiveness of the pigtail probe in repairing canalicular lacerations [11, 19, 20].

In the current study, all the cases were of grade 0 in which the lower punctum cannot be detected even on slit-lamp examination. So, we used the round-tipped, eyed pigtail probe to locate the position of the occluded lower punctum. In this way, we avoid any undue manipulation in the lower eyelid which would complicate the case. We incised upon the tented area over the tip of the pigtail probe. Then, we confirmed the patency of the rest of the lacrimal passages by syringing through the incised opening of the lower punctum.

Some reports were concerned about the potential iatrogenic damage to the normal functioning canaliculus with the pigtail probe [21]. So, we preferred to put a stent in the normal upper punctum and canaliculus which were used to locate the lower punctum. The rest of the lacrimal passages were not manipulated, so there was no need to use the classic bicanalicular lacrimal tube whose ends should be retrieved and tied in the nose. Instead, we used the self-retaining bicanalicular tube which maintains the patency of the proximal lacrimal passages.

In previous studies [6, 15, 22, 23], perforated lacrimal plugs were used to keep the punctum opened in cases of punctal stenosis. This was not applicable in the current study because all our cases were of grade 0 and needed an incision to open the occluded punctum. That would not keep the perforated plugs stable in place after implantation. However, these plugs can be used after removal of the self-retaining tubes if the punctum was not well formed enough and at a risk of restenosis.

A pitfall of the current study is that our technique is not applicable in case of a partial or complete occlusion of nasolacrimal duct. A case series on 5 cases with punctal occlusion used the pigtail probe to locate and incise the occluded punctum [13]. However, they used the classic lacrimal tube to maintain patency and prevent reocclusion of the lacrimal passages. In the current work, we excluded cases with nasolacrimal duct obstruction detected by syringing. And our technique is not suitable if there is complete atresia of the lower canaliculus whether congenital or acquired or if the upper punctum was occluded as well. In these conditions, insertion of June's tube would be appropriate.

In conclusion, using the pigtail probe is effective in treatment of severe punctal stenosis. Maintaining the punctal opening and prevention of restenosis can be achieved by using self-retaining bicanalicular stent after confirmation of nasolacrimal duct patency.

## Figures and Tables

**Table 1:** Grading of punctal stenosis according to Kashkouli et al.'s score [14].

Grade	Clinical findings
0	No punctum (agenesis)
1	Papilla is covered with a membrane (difficult to recognize)
2	Less than normal size, but recognizable
3	Normal
4	Small slit (<2 mm)
5	Large slit (≤2 mm)

**Table 2:** Grading of fluorescein dye disappearance time test [15].

Grade	Dye disappearance time (min)
1	<3
2	3–5
3	>5

**Table 3:** Munk score [16].

Grade	Clinical finding
0	No epiphora
1	Occasional epiphora requiring drying or dabbing less than twice a day
2	Epiphora requiring dabbing two to four times per day
3	Epiphora requiring dabbing five to ten times per day
4	Epiphora requiring dabbing more than ten times daily or constant tearing

**Table 4:** Comparison of preoperative and postoperative epiphora.

	Grade					
	0, *N* (%)	1, *N* (%)	2, *N* (%)	3, *N* (%)	4, *N* (%)	
Preoperative	—	—	—	9 (37.5)	15 (65.5)	
6 months (P.O.)	18 (75)	3 (12.5)	1 (4.2)	2 (8.3)	—	*t* = 16.2, *p*_1_ < 0.001
12 months (6 months after removal of the tube)	16 (66.7)	4 (16.7)	2 (8.3)	2 (8.3)	—	*t* = 15.6, *p*_2_ < 0.001

P.O., postoperative; *p*_1_, preoperative vs 6 months postoperative; *p*_2_, preoperative vs 12 months postoperative.

**Table 5:** Comparison of preoperative and postoperative fluorescein disappearance time.

	Grade			
	1, *N* (%)	2, *N* (%)	3, *N* (%)	
Preoperative	3 (12.5)	2 (8.3)	19 (79.2)	
6 months (postoperative)	21 (87.5)	3 (12.5)	—	*t* = 10.5, *p*_1_ < 0.001
12 months (postoperative) (6 months after removal of the plug)	20 (83.3)	4 (16.7)	—	*t* = 10.2, *p*_2_ < 0.001

*p*_1_, preoperative vs 6 months postoperative; *p*_2_, preoperative vs 12 months postoperative.

**Table 6:** Comparison of preoperative and postoperative Kashkouli grading of study patients.

	Grade					
	0, *N* (%)	1, *N* (%)	2, *N* (%)	3, *N* (%)	4, *N* (%)	5, *N* (%)
Preoperative	24 (100)	—	—	—	—	—
6 months (P.O.)	—	—	2 (8.3)	15 (62.5)	3 (12.5)	4 (16.7)
12 months (6 months after removal of the tube)	—	1 (4.2)	2 (8.3)	14 (58.3)	5 (20.8)	2 (8.3)

P.O., postoperative.

## Data Availability

The data used to support the findings of this study are available from the corresponding author upon request.
